# Deep Sequencing of Small RNA Repertoires in Mice Reveals Metabolic Disorders-Associated Hepatic miRNAs

**DOI:** 10.1371/journal.pone.0080774

**Published:** 2013-11-15

**Authors:** Tingming Liang, Chang Liu, Zhenchao Ye

**Affiliations:** Jiangsu Key Laboratory for Molecular and Medical Biotechnology, College of Life Science, Nanjing Normal University, Nanjing, Jiangsu, China; Huazhong University of Science and Technology, China

## Abstract

Obesity and associated metabolic disorders contribute importantly to the metabolic syndrome. On the other hand, microRNAs (miRNAs) are a class of small non-coding RNAs that repress target gene expression by inducing mRNA degradation and/or translation repression. Dysregulation of specific miRNAs in obesity may influence energy metabolism and cause insulin resistance, which leads to dyslipidemia, steatosis hepatis and type 2 diabetes. In the present study, we comprehensively analyzed and validated dysregulated miRNAs in *ob/ob* mouse liver, as well as miRNA groups based on miRNA gene cluster and gene family by using deep sequencing miRNA datasets. We found that over 13.8% of the total analyzed miRNAs were dysregulated, of which 37 miRNA species showed significantly differential expression. Further RT-qPCR analysis in some selected miRNAs validated the similar expression patterns observed in deep sequencing. Interestingly, we found that miRNA gene cluster and family always showed consistent dysregulation patterns in *ob/ob* mouse liver, although they had various enrichment levels. Functional enrichment analysis revealed the versatile physiological roles (over six signal pathways and five human diseases) of these miRNAs. Biological studies indicated that overexpression of miR-126 or inhibition of miR-24 in AML-12 cells attenuated free fatty acids-induced fat accumulation. Taken together, our data strongly suggest that obesity and metabolic disturbance are tightly associated with functional miRNAs. We also identified hepatic miRNA candidates serving as potential biomarkers for the diagnose of the metabolic syndrome.

## Introduction

MicroRNAs (miRNAs) are a class of small non-coding RNAs that repress target gene expression by a combination of mRNA degradation and translation inhibition [1]. Extensive studies have revealed important and multiple roles of miRNAs in various biological processes, such as cell proliferation [2,3], differentiation [3], death and tumorigenesis [4,5]. Furthermore, emerging evidence also indicates that miRNAs are involved in many metabolic pathways, including adipocyte differentiation, hepatic metabolic integration, insulin resistance and appetite regulation [6-8]. 

Obesity and associated metabolic disorders conditions such as insulin resistance, type 2 diabetes (T2D), hypertension, dyslipidemia, and steatosis hepatis, are worldwide epidemic, and represent major challenges for basic medical science and clinical research. Obesity is also a crucial risk factor for cardiometabolic diseases [9]. Monogenic obesity model (e.g. leptin-deficient *ob/ob* mice) exhibits overweight, hyperphagia, glucose intolerance and insulin resistance, which serves as a good biomedical model for T2D and metabolic syndrome [10,11]. On the other hand, numerous studies have deciphered that miRNAs are critical regulators of metabolism. For example, the role of miRNAs in lipid metabolism was reported in Drosophila, where deletion of mir-14 increased levels of triacylglycerol and diacylglycerol [12]. miR-122 is a liver-specific miRNA which has been identified to play roles in hepatitis C virus infection [13], cholesterol metabolism [14] and hepatocellular carcinoma [15]. Other metabolic relevant miRNAs, such as miR‑103 and miR‑107, regulate insulin sensitivity and glucose homeostasis [16]. Interestingly, hepatic miR‑34a expression is increased from steatosis to less- and more-severe nonalcoholic steatohepatitis (NASH) [17]. Jordan et al. [18] reported that transgenic over-expression of miR-143 in mice impairs insulin-stimulated AKT activation and glucose homeostasis. Therefore, dysregulation of miRNAs may contribute importantly to the metabolic abnormalities.

High-throughput sequencing of miRNAs provides a highly quantitative evaluation of known individual miRNA species [19]. Since the liver plays a central role in glucose and lipid metabolism, we attempted to identify dysregulated miRNAs by sequencing small RNAs in *ob/ob* mouse liver using Illumina/Solexa sequencing platform. The hepatic miRNA candidates under metabolic disorder conditions were validated using RT-qPCR method. We then analyzed the expression of miRNA groups at the miRNA gene cluster and gene family levels. The functional enrichment analysis of the differential miRNAs was also performed to understand their potential physiological roles. 

## Results

### Overview of miRNA Expression Profiles in ob/ob Mouse Liver

**Figure 1:**
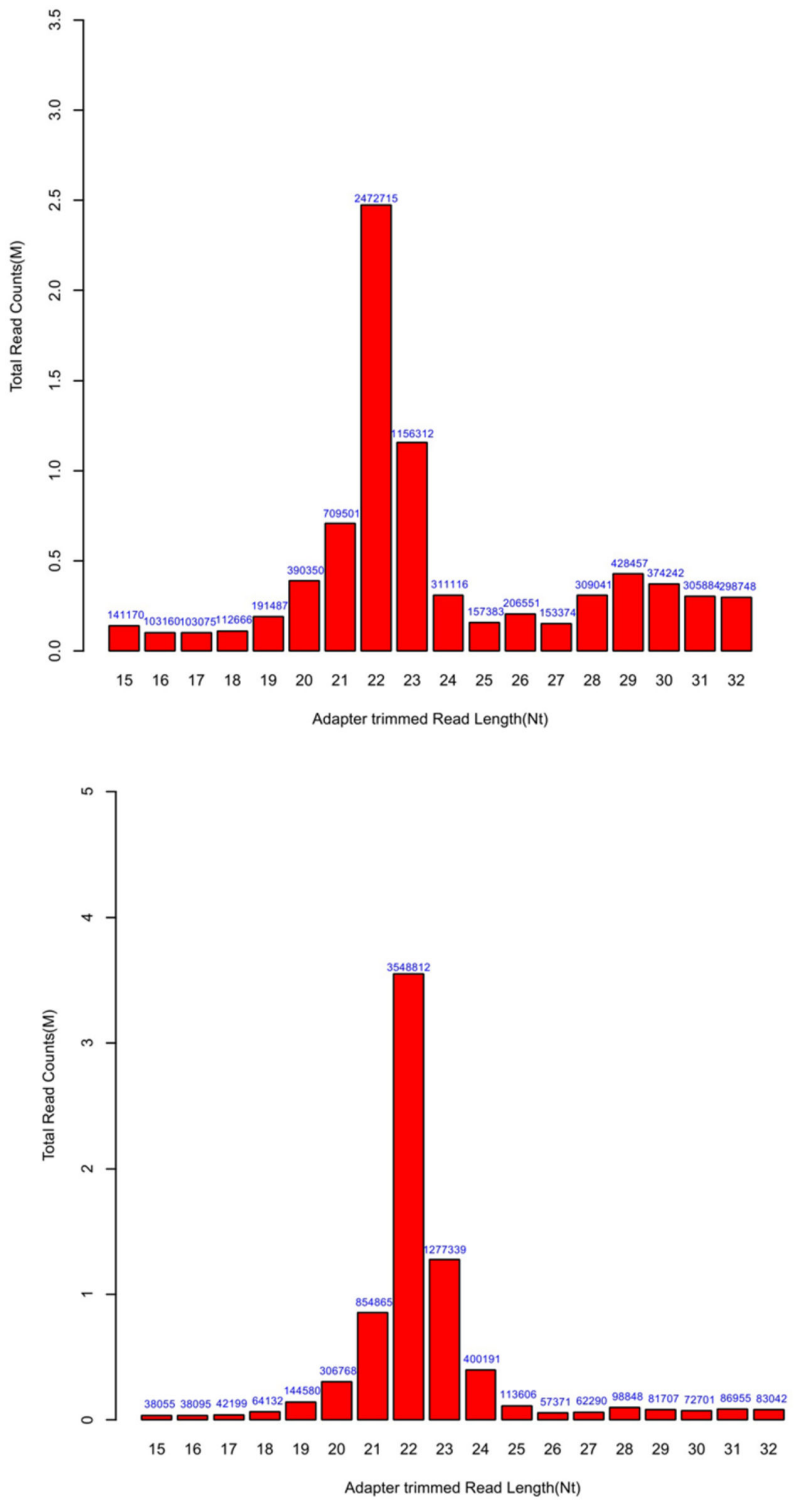
Length distributions of reads. A: The data of *ob/ob* mouse liver samples. B: The data of wild-type mouse liver samples.

**Figure 2:**
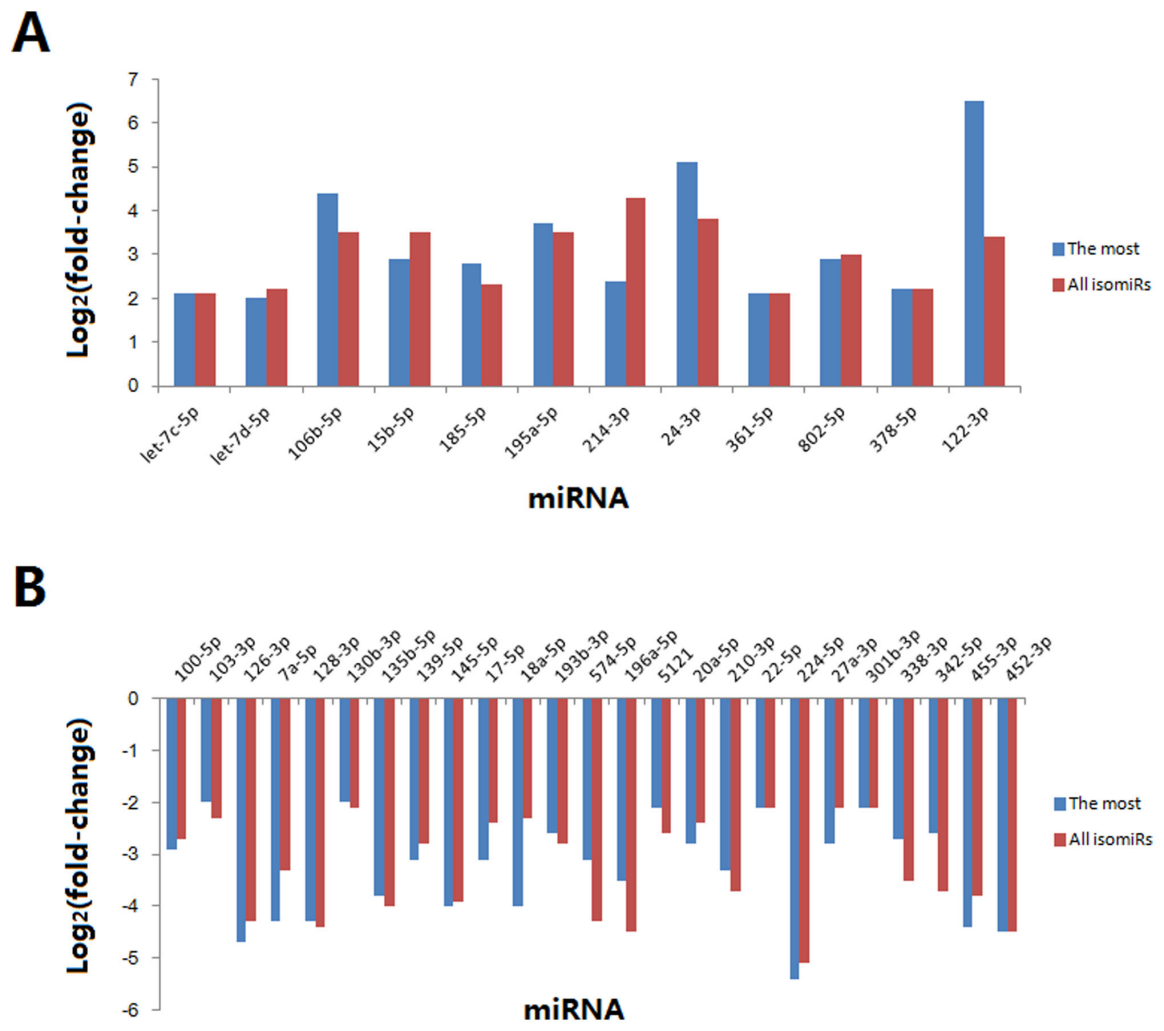
The abundance of miRNAs in *ob/ob* mouse liver. These miRNAs are abundantly expressed in *ob/ob* mouse liver *vs*. WT mouse liver (> 13.8% in the total). They are estimated based on the most abundant isomiR and sum of all isomiRs, respectively. A: up-regulated miRNAs, B: down-regulated miRNAs.

To examine the difference of miRNA expression between *ob/ob* and WT mouse liver, we used the Illumina miRNA expression profiling assay. According to short RNAs that could be mapped to mouse miRNA precursors, the most abundant length was 22 nt, as expected (Figure 1). 510 miRNAs were detected, and over 13.8% of which showed dysregulated expression. Using a 2-fold expression difference as a cutoff, 37 miRNAs showed significantly differential expression in *ob/ob* mouse liver. Among which, 12 were up-regulated and 25 were down-regulated (Figure 2). For those up-regulated, the top ten were miR-122, 24, 195a, 106b, 15b, 802, 185, 214, 378, and let-7c. miR-122 expression was most robustly dysregulated (> 6-fold) in *ob/ob* mouse liver (Figure 2A). In contrast, for those down-regulated, the top ten were miR-224, 126, 7a, 128, 455, 452, 135b, 145, 18a and 196a (Figure 2B). miR-224 expression decreased most (< -5-fold). Furthermore, multiple isomiRs (miRNA variants) have been shown to be detected from the miRNA locus due to imprecise and alternative cleavage of Drosha and Dicer. Herein, assessed fold change (log_2_) based on the different selection of isomiRs (the most abundant isomiR and sum of all isomiRs) showed various values (Table S1).

### Differentially Expressed miRNA Gene Cluster and Gene Family

**Figure 3:**
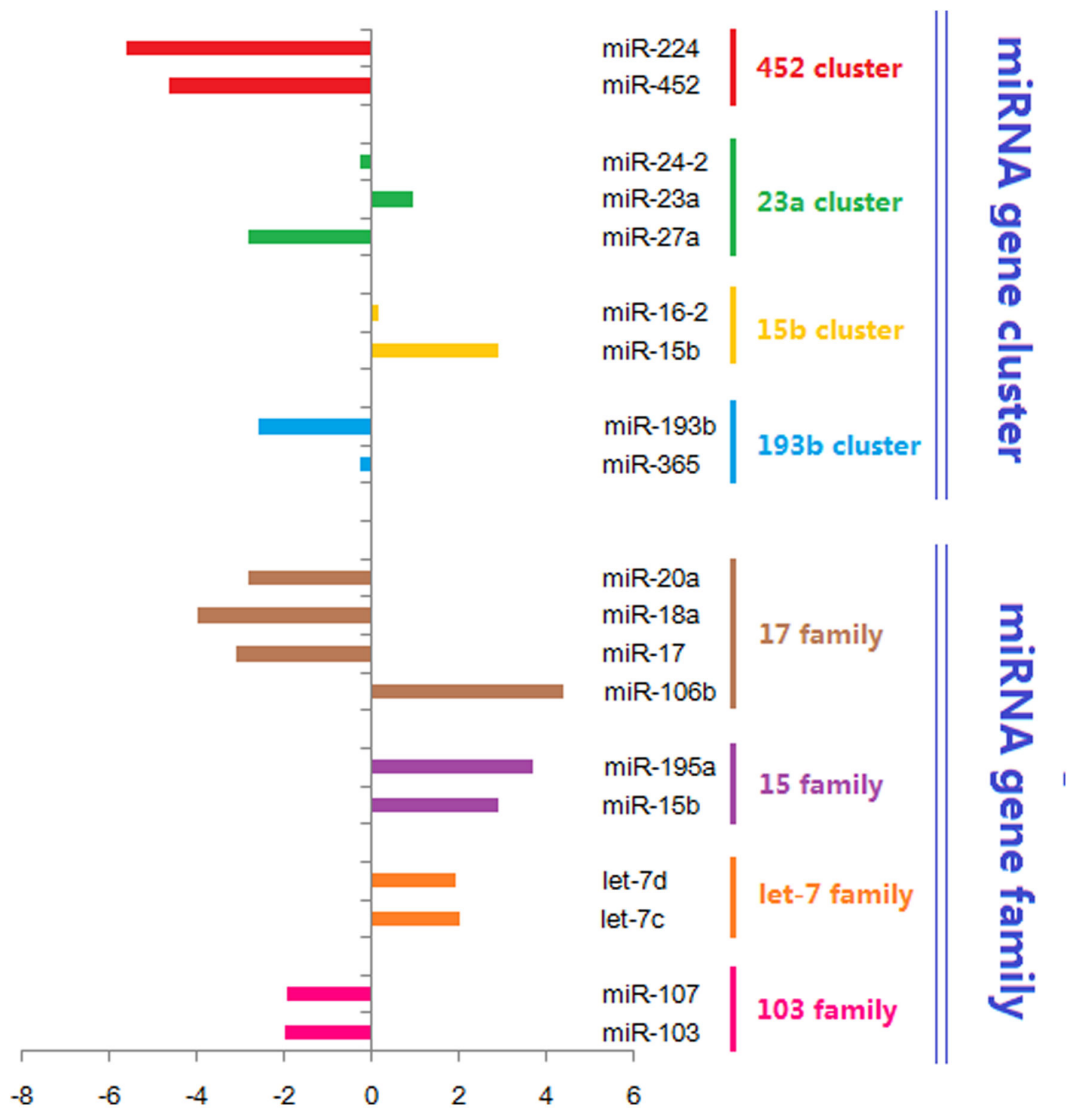
Examples of aberrantly expressed miRNA gene clusters and families based on sum of all isomiRs in *ob/ob* mouse liver.

Next, we performed a comprehensive analysis of isomiR expression patterns in miRNA gene clusters and families [20]. Four abundantly expressed miRNA gene clusters and four gene families were selected. Various enrichment levels could be found among members in miRNA gene cluster and gene family based on the original sequence counts (Figure 3). Clustered miRNAs and homologous miRNAs had various expression levels (even involved in larger expression divergence), but they always showed consistent dysregulation patterns (For example, mir-15b cluster and mir-193b cluster; mir-15 family) although the fold change may differ. 

### Functional Enrichment Analysis

Functional enrichment analysis of dysregulated miRNAs suggested that the versatile biological roles (Table 1) of these miRNAs were included. These miRNAs contributed to many essential biological processes, such as TGF-beta signaling pathway, cell cycle, Wnt signaling pathway, MAPK signaling pathway, ErbB signaling pathway, Jak-STAT signaling pathway, and etc. Furthermore, these aberrantly expressed miRNA species were involved in occurrence and development of some human diseases, including chronic myeloid leukemia, pancreatic cancer, colorectal cancer, glioma, prostate cancer, and etc.

**Table 1:** Enrichment pathway analysis based on experimentally validated target mRNAs.

**Pathway**	**Gene Numbers**	**EnrichmentP-value**	**Target Genes**
Chronic myeloid leukemia	11	2.81E-23	Acvr1b;Ccnd1;Cdk6;Cdkn1b;E2f3;Myc; Pik3r2;Runx1;Smad3;Smad4;Tgfbr1
Pancreatic cancer	9	1.00E-18	Acvr1b;Ccnd1;Cdk6;E2f3;Pik3r2;Smad3; Smad4;Stat3;Tgfbr1
Colorectal cancer	8	1.54E-15	Acvr1b;Ccnd1;Myc;Pik3r2;Smad3;Smad4; Tcf7l2;Tgfbr1
Acute myeloid leukemia	7	8.53E-15	Ccnd1;Kit;Myc;Pik3r2;Runx1;Stat3;Tcf7l2
TGF-beta signaling pathway	7	3.08E-13	Acvr1b;Myc;Rbl2;Smad3;Smad4;Smad5;Tgfbr1
Cell cycle	7	1.95E-12	Ccnd1;Cdk6;Cdkn1b;E2f3;Rbl2;Smad3; Smad4
Wnt signaling pathway	7	8.77E-12	Camk2d;Camk2b;Ccnd1;Myc;Smad3;Smad4;Tcf7l2
Glioma	6	5.55E-12	Camk2d;Camk2b;Ccnd1;Cdk6;E2f3;Pik3r2
Prostate cancer	6	3.58E-11	Ccnd1;Cdkn1b;E2f3;Foxo1;Pik3r2;Tcf7l2
Small cell lung cancer	6	4.08E-11	Ccnd1;Cdk6;Cdkn1b;E2f3;Myc;Pik3r2
MAPK signaling pathway	6	2.01E-08	Acvr1b;Bdnf;Mapk14;Mef2c;Myc;Tgfbr1
Adherens junction	5	1.63E-09	Acvr1b;Smad3;Smad4;Tcf7l2;Tgfbr1
ErbB signaling pathway	5	3.44E-09	Camk2d;Camk2b;Cdkn1b;Myc;Pik3r2
Jak-STAT signaling pathway	5	5.94E-08	Ccnd1;Myc;Pik3r2;Spred1;Stat3

### Validation of the Differentially Expressed miRNAs by RT-qPCR

**Figure 4:**
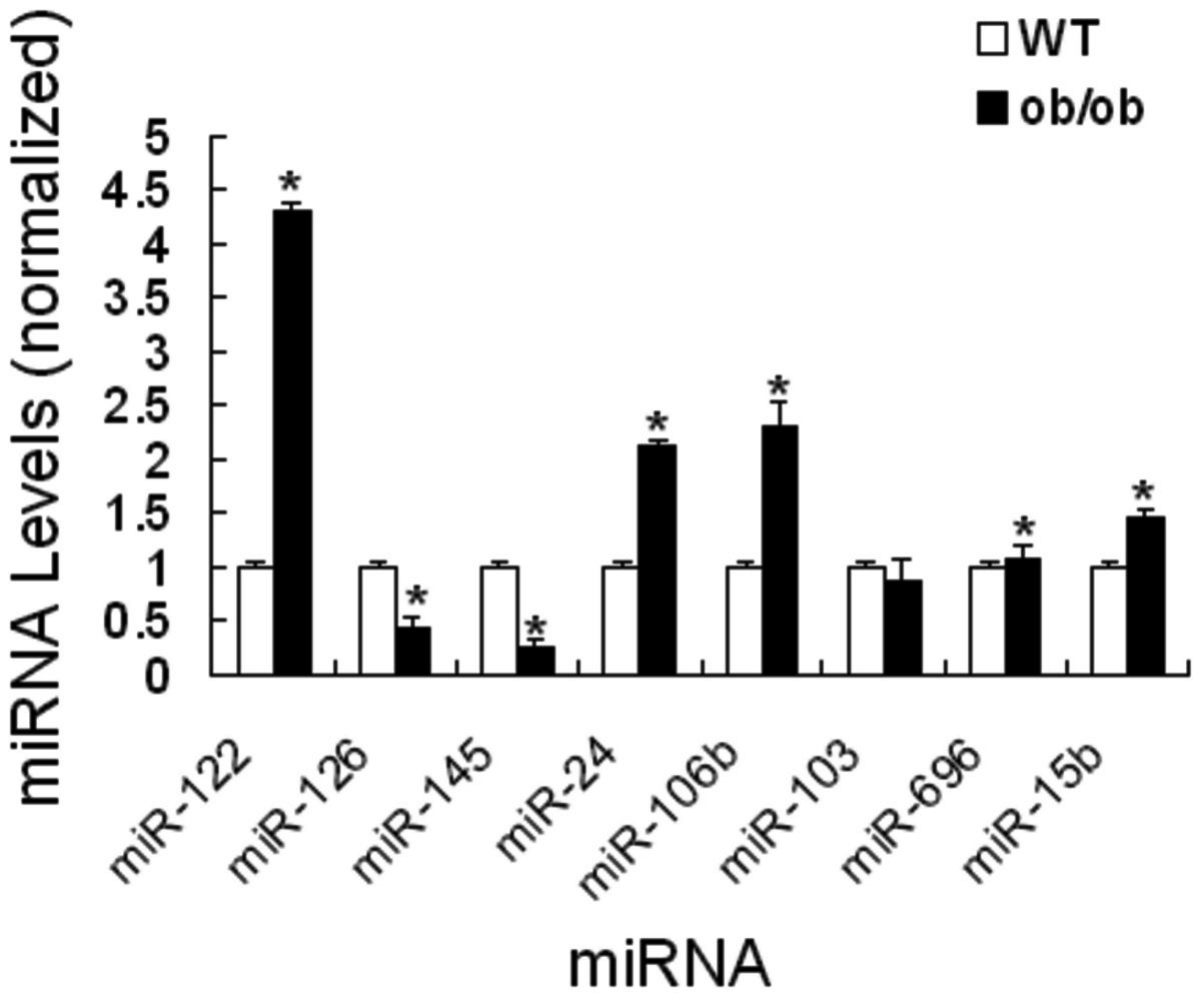
Validation of the differential expression pattern of miRNA species by RT-qPCR analysis. RT-qPCR analysis was performed to quantify the expression levels of miR-122, 126, 145, 24, 106b, 103, 696 and 15b.

To further validate the abnormal expression of the collected miRNA species, we selected several typical miRNAs and performed RT-qPCR for double-check. As expected, the result showed similar expression patterns as observed in deep sequencing. For example, miR-122, 24, 106b, 696 and 15b were up-regulated, but miR-126, 145 and 103 were down-regulated (Figure 4). 

### miR-126 or miR-24 Regulates Lipid Accumulation in AML12 Hepatocytes Exposed to FFAs

**Figure 5:**
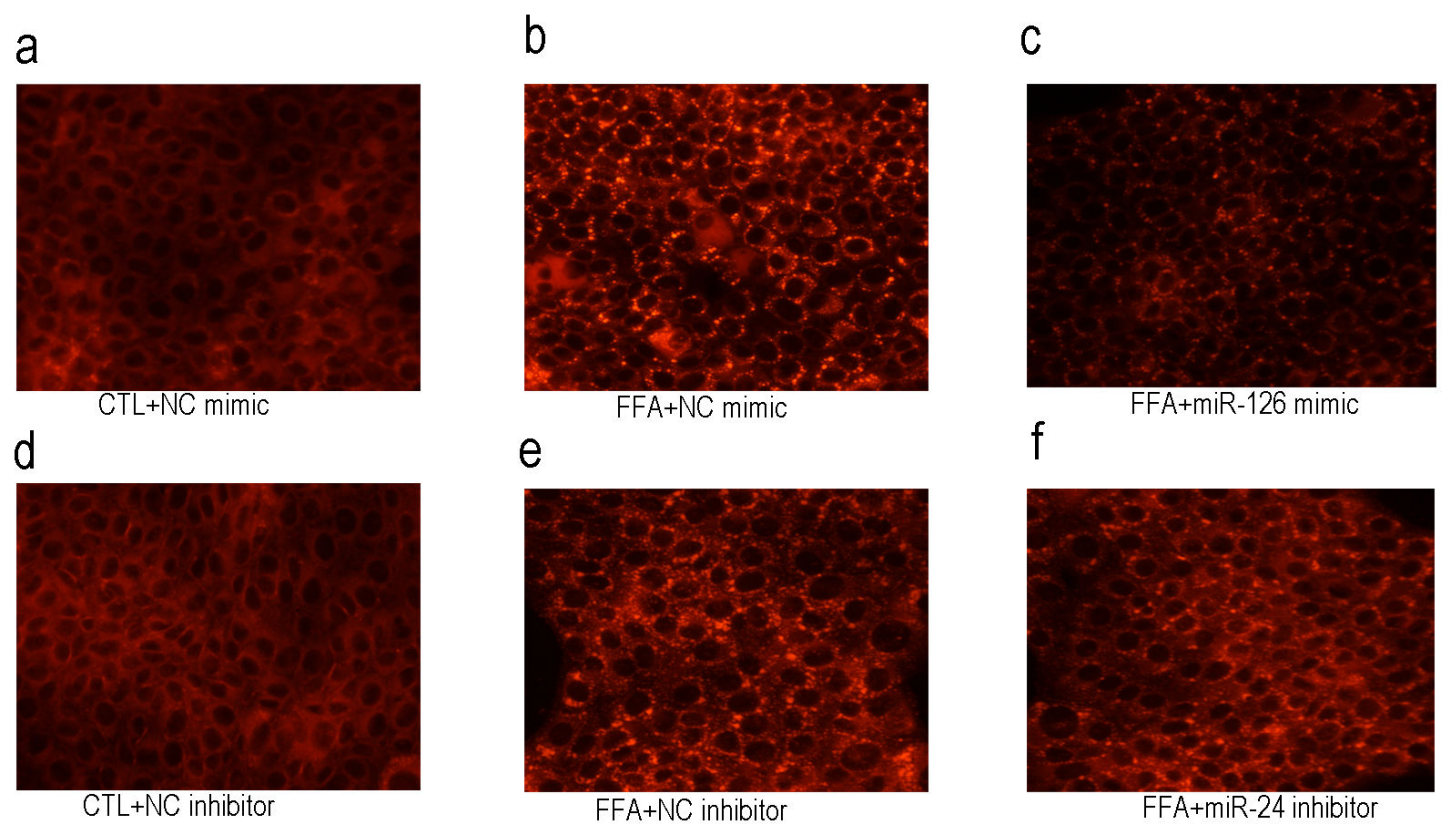
Overexpression of miR-126 or inhibition of miR-24 antagonizes FFAs-induced lipid accumulation in AML12 hepatocytes. AML12 cells were transfected with either miR-126 mimic, miR-24 inhibitor or their corresponding negative controls. 48 hs after transfection, FFAs (0.4 mM) was added and cells were incubated for another 18 hs. Intracellular lipid levels were measured by Nile red staining.

 To determine whether the disordered miR-126 or miR-24 levels induced by free fat acid (FFA) affect cellular triglyceride (TG) accumulation, we examined the TG levels in AML-12 cells transfected with mimic (Negative control) NC, miR-126 mimic or inhibitor NC, miR-24 inhibitor using Nile red staining. FFA significantly increased the TG accumulation in AML-12 cells compared with controls (Figure 5). More important, overexpression of miR-126 or inhibition of miR-24 markedly improved fat accumulation in AML-12 cells expose to FFA (Figure 5). 

## Discussion

Dysregulation of miRNA expression has been shown to influence glucose homeostasis, cholesterol metabolism and cause insulin resistence, which thus plays an important role in the pathogenesis of metabolic disorders such as T2D, atherosclerosis, fatty liver and Alzheimer’s disease [21,22]. In the present study, deep sequencing of small RNA populations in *ob/ob* mice reveals metabolic disorders-associated hepatic miRNAs. 

Here, we observed that 37 metabolic disorder-associated hepatic miRNAs were differentially expressed in *ob/ob* mouse liver, and they regulated the expression of several important downstream target genes. Overview of the miRNA expression profiles in *ob/ob* mouse liver, miR-122 is the most abundant miRNA (Figure 2). It has been demonstrated that miR-370 induces the accumulation of hepatic triglycerides through interacting with miR-122 and Cpt 1α [23]. Another study showed that hepatocytes from anti-miR-122-treated mice showed increased fatty acid oxidation rates and reduced fatty acid synthesis. Accordingly, antagonist of miR-122 used in diet-induced obese mice significantly improved hepatic steatosis and reduced levels of triglyceride accumulation. Taken these previous observations and our data, miR-122 is a risk factor to induce obesity and hepatic metabolic dysfunction. On the other hand, miR-126 is known as an endothelium-specific miRNA, and has been reported to promote angiogenesis by targeting SPRED1 to inhibit VEGF signaling [24]. It also acts as an oncogene by targeting SOX2 in gastric cancer cells [25]. Our results validated that miR-126 was down-regulated in *ob/ob* mouse liver. Similarly, miR‑33 was another down-regulated miRNA in *ob/ob* mouse live. Silencing of miR-33 *in vivo* increases hepatic expression of ABCA1 and SREBP-2 [26,27], leading to dysregulation of cholesterol homeostasis. Thus, miR-33 is critical to maintain normal cholesterol metabolism. A recent study suggested that miR-27b was responsive to lipid levels and regulated several key lipid-metabolism genes during dyslipidemia [28]. This miRNA was also detected to be decreased in *ob/ob* mouse liver, suggesting the impaired responsiveness to fatty acids under this pathophysiological condition. Last but not least, miR-106b was very interesting and it decreased ABCA1 levels and impairs cellular cholesterol homeostasis in neuronal cells [29]. Its function may extend to the liver system according to our data. Collectively, the abnormally expressed miRNAs in *ob/ob* livers were involved in lipid synthesis, fatty acid oxidation and cholesterol homeostasis through their target genes. 

Most miRNA studies focus on finding targets of individual miRNAs, yet half of the total miRNAs are co-expressed as clusters [30]. The expression pattern of miRNA gene clusters and gene families in *ob/ob* mouse liver showed that these miRNAs were coordinately regulated (Figure 3). The miR-15b/16-2 cluster generates miR-15b/15b* and 16-2/ 16*-2. Both miR-15b and miR-16-2 were over-expressed in *ob/ob* mouse liver, however, they were down-regulated in SGC790/VR cells [31]. On the other hand, miR-103 family (including miR-103 and miR-107) was dysregulated in *ob/ob* mouse liver. These results demonstrate that the miRNA gene family coordinate interaction with each other and function together in the pathogenesis of liver metabolic disorders with consistent dysregulation patterns.

Functional enrichment analysis showed that over six signal pathways and five human diseases were involved. In addition, miRNAs may serve as nodes cross-linking various signaling pathways by the integration of transcriptional inputs or by their functional regulatory outputs, thus highlighting the potential important roles in the epigenetic regulation of lipid metabolic abnormalities. The small non-coding regulatory molecules have opened new avenues for the treatment of metabolic diseases. 

The functional analysis in AML-12 liver cells showed that dysregulation of miR-126 and miR-24 is correlated with fat accumulation (Figure 5). In recent years, several miRNAs, such as miR-155 [32], and miR-217 [33], related fat liver disease have been identified. Although our present study supports the concept that miRNAs contribute to the fat accumulation in mice cell, further work is warranted to identify more specific hepatic miRNAs and their functions involved in lipid metabolic disorders. Whether up-regulation of reduced hepatic miR-126 using miR-126 mimic (or down-regulation of elevated hepatic miR-24 using antagomiR-24) approaches would alleviate liver steatosis in high fat diet-fed mice is currently under investigation in our laboratory.

In conclusion, the present study demonstrated that various miRNAs were differentially expressed in *ob/ob* mouse liver (especially for miR-126 and miR-24), suggesting that they were tightly linked to obesity and other metabolic disorders. The candidate miRNAs we identified may be potential biomarkers for the diagnose of the metabolic syndrome.

## Materials and Methods

### Animal Studies

Male obese ob/ob mice on a C57BL/6J background (12 weeks old) and their male WT littermates were purchased from the Model Animal Research Center of Nanjing University (Nanjing, China), and were housed and maintained in a 12-h light/12-h dark cycle (light/dark, 12:12) in a temperature- and humidity-controlled environment. All protocols complied with, and all animals received humane care according to the criteria outlined in the NIH ‘Guide for the Care and Use of Laboratory Animals’ and the approved regulations set by the Laboratory Animal Care Committee at Nanjing Normal University. 

### RNA Isolation, Construction of Small RNA Library and High-Throughput Small RNA Sequencing

Five liver samples from *ob/ob* or WT mice (20 mg each in weight) were equally pooled together and kept in RNAfixer (BioTeke Co. Ltd, Beijing, China) at -70 °C until use. 

RNA with low molecular weight from liver was extracted using a mirVana™ miRNA isolation kit (Ambion, Austin, TX, USA) following the manufacturer’s protocol. The purity and concentration of RNA samples were determined with NanoDrop ND-1000 spectrophotometer (Nano Drop Technologies, Wilmington, DE, USA) at 260/280 nm (ratio > 2.0). 60 μg of total RNA from the pooled samples was used for library preparation and sequencing. The libraries were used for the Illumina/Solexa miRNA deep sequencing which included the following steps: total RNA of each sample was sequentially ligated to 3’ and 5’ small RNA adapters. cDNA was then synthesized and amplified using Illumina’s proprietary RT primers and amplification primers, respectively. Subsequently, PCR amplified fragments within 110-130 bp were extracted and purified from the PAGE gel. When the completed libraries were quantified with Agilent 2100 Bioanalyzer, the DNA fragments in the libraries were denatured with 0.1M NaOH to generate single-stranded DNA molecules, which were captured on Illumina flow cells, amplified *in situ* and finally sequenced for 36 cycles on Illumina’s Genome Analyzer IIx according to the vendor’s recommended protocol (Illumina, San Diego, CA, USA) [34-36].

Data deposition: The data reported in this paper have been deposited in the Sequence Read Archive (SRA) database, www.ncbi.nlm.nih.gov/sra (accession no. SRR987316; SRR987317)

### Bioinformatic Analysis of Solexa Sequencing Data

Low-quality sequence reads failed to pass the quality filter were removed according to the criteria of Solexa/Illumina. 3’ adapter sequences were trimmed from clean reads and the tags shorter than 15nt were discarded. Then reads were aligned to the latest known mouse reference miRNA precursor set (Sanger miRBase 19.0, http://www.mirbase.org/) [37-39] using Novoalign software (v2.07.11). In this aspect, reads (counts < 2) were discarded when calculating the miRNA expression. In order to characterize the isomiR variability, sequences matching the miRNA precursors in the mature miRNAs region ± 4 nt (no more than 1 mismatch) were accepted as mature miRNA isomiRs, which were grouped according to the 5-prime (5p) or 3-prime (3p) arm of the precursor hairpin [40]. 

The relative abundance of miRNAs was estimated by the number of reads for each miRNA sequenced, and the read amount of each unique sequence was normalized to reads per million according to the total read count of the mapped reads data set [41]. To compare the differential expression between *ob/ob* and WT mice, the log_2_ ratio of miRNA (*ob/ob*)/miRNA (WT) was used, and miRNAs exhibiting a fold change value (log_2_) of higher than 2 or lower than -2 in expression were considered differentially expressed. 

The differentially expressed miRNAs were selected to query for their Gene Ontology Enrichment categories using the CapitalBio Molecule Annotation System V4.0 (MAS, http://bioinfo.capitalbio.com/mas3/). The target mRNAs of these miRNAs were determined based on known experimentally validated target mRNAs from the miRTarBase database [42], the miRecords database [43] and the TarBase database [44]. If no validated target was identified in these databases, the prediction software of TargetScan [45] was used to predict target mRNAs.

### RT-qPCR Validation

To confirm the expression of miRNAs identified by the deep sequencing approach, RT-qPCR analysis was performed using the total RNA samples from five *ob/ob* or WT mouse livers. For miRNA quantification, Bulge-loop™ miRNA RT-qPCR Primer Sets (one RT primer and a pair of qPCR primers for each set) specific for miR-106b, 24, 122, 126, 103, 696, 15b and 145 were designed by RiboBio (Guangzhou, China). The total RNA was extracted with TRIzol Reagent (Invitrogen Corp., Carlsbad, CA). miRNA bulge-loop was then reverse transcribed with the PrimeScript RT reagent kit (Takara Bio, Tokyo, Japan) and quantified by qPCR using SYBR Premix Ex TaqTM II (Takara Bio, Tokyo, Japan) according to the indicated manufacturer's instructions. miRNA expression was normalized to snRNA U6 to get the relative abundance. Averages of three independent experiments each performed in triplicate with standard errors were presented.

### Cell Culture and Treatments

The mouse AML12 hepatocyte cells were obtained from American Type Culture Collection and were cultured in DMEM/F12 (Wisent, Nanjing) containing 10% heat-inactivated fetal bovine serum (Wisent, Nanjing) and 1% penicillin-streptomycin (Invitrogen). To test the biological roles of miR-126 and miR-24, miR-126 mimic, miR-24 inhibitor or their corresponding negative controls were transfected into AML12 cells using lipofectamine 2000 reagent (Invitrogen) according to the manufacturer’s protocol. After 48 hr, intracellular lipid accumulation was induced according to previously described modified methods [46] and [47]. Briefly, bovine serum albumin (BSA)-conjugated free fatty acids (FFAs; oleate/palmitate, 1/1, v/v) were mixed, and then the culture medium were added into mixture to give a final concentration of 0.4 mM. Control cell cultures were incubated with equal amount of BSA. The stimulation of FFAs lasted for 18 hr.

### Nile Red Staining

Cell were fixed with 4% formaldehyde and stained with Nile Red solution (1µg/ml) for 10 min at 37 °C. Lipid-bound Nile Red fluorescence was observed with a fluorescence microscope (*Ti-S*, Nikon).

### Statistical Analysis

Data are presented as the mean ± S.E. Unless otherwise indicated, the statistics was performed using Student’s *t*-test when only two groups were compared. Results with a *P* < 0.05 were considered statistically significant.

## Supporting Information

Table S1 **Various fold change values (log_2_) based on different selection of miRNA sequences.**
(DOC)
