# Supplementary material for: Deep Sequencing of Small RNA Repertoires in Mice Reveals Metabolic Disorders-Associated Hepatic miRNAs
Source: PLoS One. 2013 Nov 15;8(11):e80774. doi: 10.1371/journal.pone.0080774 (PMC3829963; doi:10.1371/journal.pone.0080774)
Supplement: Table S1 — Various fold change values (log2) based on different selection of miRNA sequences. (DOC) [file pone.0080774.s001.doc]

**Supplementary files**

**Table S1 Various fold change values (log2) based on different selection of miRNA sequences.** The-most indicates the most abundant and dominant isomiR sequence; All-isomiRs indicates sum of all the isomiRs.

| **MiRNA** | **Chr** | **Fold-change**  **(the most)** | **Fold-change**  **(all isomiRs)** | **Cluster** | **Family** | **Up/**  **Down** |
| --- | --- | --- | --- | --- | --- | --- |
| let-7c-5p | 16 | 2.1 | 2.1 | miR-99a | let-7 | Up |
| let-7d-5p | 13 | 2.0 | 2.2 | miR-7d/7a-1/7f-1 | let-7 | Up |
| miR-106b-5p | 5 | 4.4 | 3.5 | miR-106b/93/25 | mir-17 | Up |
| miR-15b-5p | 3 | 2.9 | 3.5 | miR-15b/16-2 | mir-15 | Up |
| miR-185-5p | 16 | 2.8 | 2.3 | - | mir-185 | Up |
| miR-195a-5p | 11 | 3.7 | 3.5 | miR-195a/497 | mir-15 | Up |
| miR-214-3p | 1 | 2.4 | 4.3 | miR-199a-2/214 | mir-214 | Up |
| miR-24-3p | 13/8 | 5.1 | 3.8 | miR-23b/27b/3074-1/3074-2/24-1/24-2 | mir-24 | Up |
| miR-361-5p | X | 2.1 | 2.1 | - | mir-361 | Up |
| miR-802-5p | 16 | 2.9 | 3.0 | - | mir-802 | Up |
| miR-378-5p | 18/11/14/10 | 2.2 | 2.2 | - | - | Up |
| miR-122-3p | 18 | 6.5 | 3.4 | - | mir-122 | Up |
| miR-100-5p | 9 | -2.9 | -2.7 | - | mir-99 | Down |
| miR-103-3p | 11/2 | -2.0 | -2.3 | - | mir-103 | Down |
| miR-126-3p | 2 | -4.7 | -4.3 | - | mir-126 | Down |
| miR-7a-5p | 13/7 | -4.3 | -3.3 | - | mir-7 | Down |
| miR-128-3p | 1/9 | -4.3 | -4.4 | - | mir-128 | Down |
| miR-130b-3p | 16 | -2.0 | -2.1 | - | mir-130 | Down |
| miR-135b-5p | 1 | -3.8 | -4.0 | - | mir-135 | Down |
| miR-139-5p | 7 | -3.1 | -2.8 | - | mir-139 | Down |
| miR-145-5p | 18 | -4.0 | -3.9 | - |  | Down |
| miR-17-5p | 14 | -3.1 | -2.4 | - | mir-17 | Down |
| miR-18a-5p | 14 | -4.0 | -2.3 | miR-17/18a/19a/20a/19b-1/92a-1 | mir-17 | Down |
| miR-193b-3p | 16 | -2.6 | -2.8 | miR-193b/365-1 | mir-193 | down |
| miR-574-5p | 5 | -3.1 | -4.3 | - | mir-574 | down |
| miR-196a-5p | 11/15 | -3.5 | -4.5 | - | mir-196 | down |
| miR-5121 | 7 | -2.1 | -2.6 | miR-5121/150 |  | down |
| miR-20a-5p | 14 | -2.8 | -2.4 | miR-17/18a/19a/20a/19b-1/92a-1 | mir-17 | down |
| miR-210-3p | 7 | -3.3 | -3.7 | - | mir-210 | down |
| miR-22-5p | 11 | -2.1 | -2.1 | - | mir-22 | down |
| miR-224-5p | X | -5.4 | -5.1 | miR-224/452 | mir-224 | down |
| miR-27a-3p | 8 | -2.8 | -2.1 | miR-23a/27a/24-2/3074-2 | mir-27 | down |
| miR-301b-3p | 16 | -2.1 | -2.1 | miR-301b/130b | mir-130 | down |
| miR-338-3p | 11 | -2.7 | -3.5 | miR-3065/338 | mir-338 | down |
| miR-342-5p | 12 | -2.6 | -3.7 | - | mir-342 | down |
| miR-455-3p | 4 | -4.4 | -3.8 | - | mir-455 | down |
| miR-452-3p | X | -4.5 | -4.5 | miR-452/224 | mir-452 | down |

These miRNAs are abundantly expressed species in *ob/ob* mouse liver *vs.* WT mouse liver. They are top up-regulated or down-regulated (fold change value > 2.0 or < -2.0). Chr: indicates the genomic locations on chromosome of the miRNA genes (pre-miRNAs), including multicopy pre-miRNAs. For example, genomic locations of miR-378-5p are located on chr18 (miR-378a), 11(miR-378b), 14 (miR-378c) and 10 (miR-378d). The most: indicates the most abundant isomiR; all isomiRs: indicates sum of all the isomiRs. ‘-’means there is no record in the miRBase for gene cluster or gene family.
